# Participant experiences with a multipurpose vaginal ring for HIV and pregnancy prevention during a phase 1 clinical trial: learning from users to improve acceptability

**DOI:** 10.3389/frph.2023.1147628

**Published:** 2023-07-06

**Authors:** Mary Kate Shapley-Quinn, Mei Song, Beatrice A. Chen, Brid Devlin, Ellen Luecke, Jill Brown, Diana L. Blithe, Sharon L. Achilles, Ariane van der Straten

**Affiliations:** ^1^ Women’s Global Health Imperative, RTI International, Berkeley, CA, United States; ^2^ Magee-Womens Research Institute, Pittsburgh, PA, United States; ^3^ Department of Obstetrics, Gynecology, and Reproductive Sciences, University of Pittsburgh, School of Medicine, Pittsburgh, PA, United States; ^4^ Center for Biomedical Research, Population Council, New York, NY, United States; ^5^ Contraceptive Development Program, Eunice Kennedy Shriver National Institute of Child Health and Human Development, Bethesda, MD, United States; ^6^ Center for AIDS Prevention Studies, Department of Medicine, University of California, San Francisco, San Francisco, CA, United States; ^7^ ASTRA Consulting, Kensington, CA, United States

**Keywords:** acceptability, multi-purpose prevention technologies, vaginal ring, HIV prevention, contraception, qualitative

## Abstract

**Introduction:**

With high concurrent global rates of HIV incidence and unintended pregnancy, there is a need to provide options beyond condoms to enable users to simultaneously prevent HIV acquisition and pregnancy. Multiple vaginal rings are in development as “MPTs” (multipurpose prevention technologies) as they are shown to provide several co-occurring benefits such as discretion, convenience, reversibility and user control.

**Methods:**

In this Phase 1 trial of a 3-month MPT ring in the U.S., 25 participants (low-risk for HIV and pregnancy) were randomized to use the study ring for 90 days continuously or in 28-day cycles with 2-day removal periods in between. All participants completed in-depth interviews at the end of their study participation.

**Results:**

Overall, the ring was well tolerated. Participants resoundingly endorsed the concept of an extended-use, dual-purpose vaginal ring, but reported too many functional challenges and side effects to endorse this particular ring. Participants assigned to the continuous regimen reported more positive experiences with ring use than those in the cyclic group. A minority of participants who experienced minimal side effects and did not experience challenges with vaginal retention of the ring found it appealing. However, the majority of participants experienced challenges (ring slippage, expulsions, side effects, vaginal bleeding changes) with product use that outweighed the potential benefits and led them to report that – in the future – they would not be interested in using this specific version of the ring in its current form. A subset expressed interest in using the current MPT ring under certain conditions (e.g., if fewer expulsions, less bleeding, higher risk for HIV/pregnancy).

**Discussion:**

User feedback regarding participant experiences and challenges with the study ring was continuously shared with the product developer, underscoring the value of early-stage end-user feedback in product development.

## Introduction

Globally, the risks of HIV and of unintended pregnancy remain high. Combining both indications into a single product, or a Multipurpose Prevention Technologies (MPT), is important and generally favored by women (1). MPTs have the potential to simplify use and access, be more cost effective, improve method framing (as a contraceptive rather than as disease prophylaxis), and therefore may increase product uptake and adherence (2–4). However, current MPT options are limited to male and female condoms. While condoms are highly effective under ideal conditions, consistent use is compromised by multiple socio-behavioral barriers (5–7). Prior research has suggested that a user-centric approach, built on understanding needs and desires of end-users, is essential to ultimately developing a successful and acceptable MPT product (8–10).

Previous research demonstrated that women highly value discretion, self-reliance, efficacy, and convenience in a prevention product (11, 12). The vaginal ring meets these criteria and was found globally to be highly acceptable as a single indication product for HIV prevention and separately, for contraception (3, 13, 14). Studies have repeatedly shown high acceptability among women who use the etonogestrel/ethinyl estradiol contraceptive ring that is woman-controlled, discreet, coitally independent (monthly dosage) while also being fully and quickly reversible (15–24). Studies of contraceptive vaginal rings have shown that the contraceptive ring (compared to products like oral pills and patches) was preferred by adolescent and adult women (18, 25). As an HIV prevention method, a monthly silicone vaginal ring releasing the antiretroviral dapivirine (DPV) was shown to be safe and effective in Phase III trials and open label extension studies (26–29), and was well accepted among women in sub-Saharan Africa (SSA) (30–32), as well as among women of various age groups in the U.S (33–36). The monthly DPV ring is currently recommended for use by the WHO for those at substantial risk for HIV (37), has been approved in multiple African countries, and is undergoing regulatory review in several other African countries. Longer duration (i.e., 3 month) HIV PrEP rings are also being assessed in clinical trials, and a recent U.S. study found that user-convenience drove preference for the 3-month ring vs. the 1-month ring (38).

MPTs in the form of injectables are most highly desired by women in the U.S and in SSA, though studies have shown a substantial minority of women would prefer vaginal methods – including rings - over injections (or willing to use vaginal methods if injections were not available) (12, 20, 39–41). This highlights the importance of developing different delivery forms for MPTs. Rings are suitable devices as MPTs, as they can be loaded with sufficient drug(s) for more than one indication and can provide an extended duration of protection (42). Much of the previous research on end-user opinions of an MPT vaginal ring was hypothetical, drawing from cross-sectional data collection activities, scenarios embedded in studies of HIV prevention products that include vaginal rings, or placebo studies (12, 19, 20, 39, 43–45). Results are available from few studies to date that include end-user experiences with active MPT vaginal rings (46–48). There are currently 12 different rings being developed as MPTs, most in preclinical or early clinical stages (49). As MPT vaginal rings enter and progress through the development pipeline, gaining an understanding of end-user preferences and acceptability during the early stages of product development and clinical trial evaluation will be essential to optimization (42, 50).

This paper describes the findings from in-depth interviews (IDIs), acceptability questionnaires, and text messages from study participants, and s used to assess acceptability of and adherence to an MPT ring for HIV and pregnancy prevention, used continuously or cyclically by low-risk women enrolled in a Phase I trial in Pittsburgh, Pennsylvania, USA. The in-depth interview data also describes participant perspectives on an “ideal” vaginal ring and MPTs in general, in addition to describing the favorable and unfavorable attributes of the study product used.

## Methods

MTN-044/IPM 053/CCN019 was an open label, Phase 1 trial conducted in Pittsburgh, Pennsylvania, USA. Participants were randomized (1:1) to one of two usage regimens for a 3-month vaginal ring developed for prevention of unintended pregnancy and HIV: continuous use for approximately 90 days or cyclic use (3 cycles each comprised of 28 days of ring usage followed by ring removal for 2 days). While the primary objectives of the trial were safety and pharmacokinetic data for the DPV/LNG ring, two exploratory objectives were to understand participant adherence to the assigned regimens and acceptability of using this ring for a dual HIV and pregnancy prevention indication.

The study took place between July 2018 and October 2019 and enrolled 25 participants who were aged 18–45 years (inclusive); assigned female sex at birth; HIV-uninfected and in general good health; and not at risk for pregnancy, defined as consistently using an effective, non-hormonal method of contraception for the duration of study participation, abstinence, or exclusively engaging in sex with individuals assigned female sex at birth. Participants were offered male condoms at each visit. Further enrollment criteria are described elsewhere (51, 52).

### Study product

**Figure 1:**
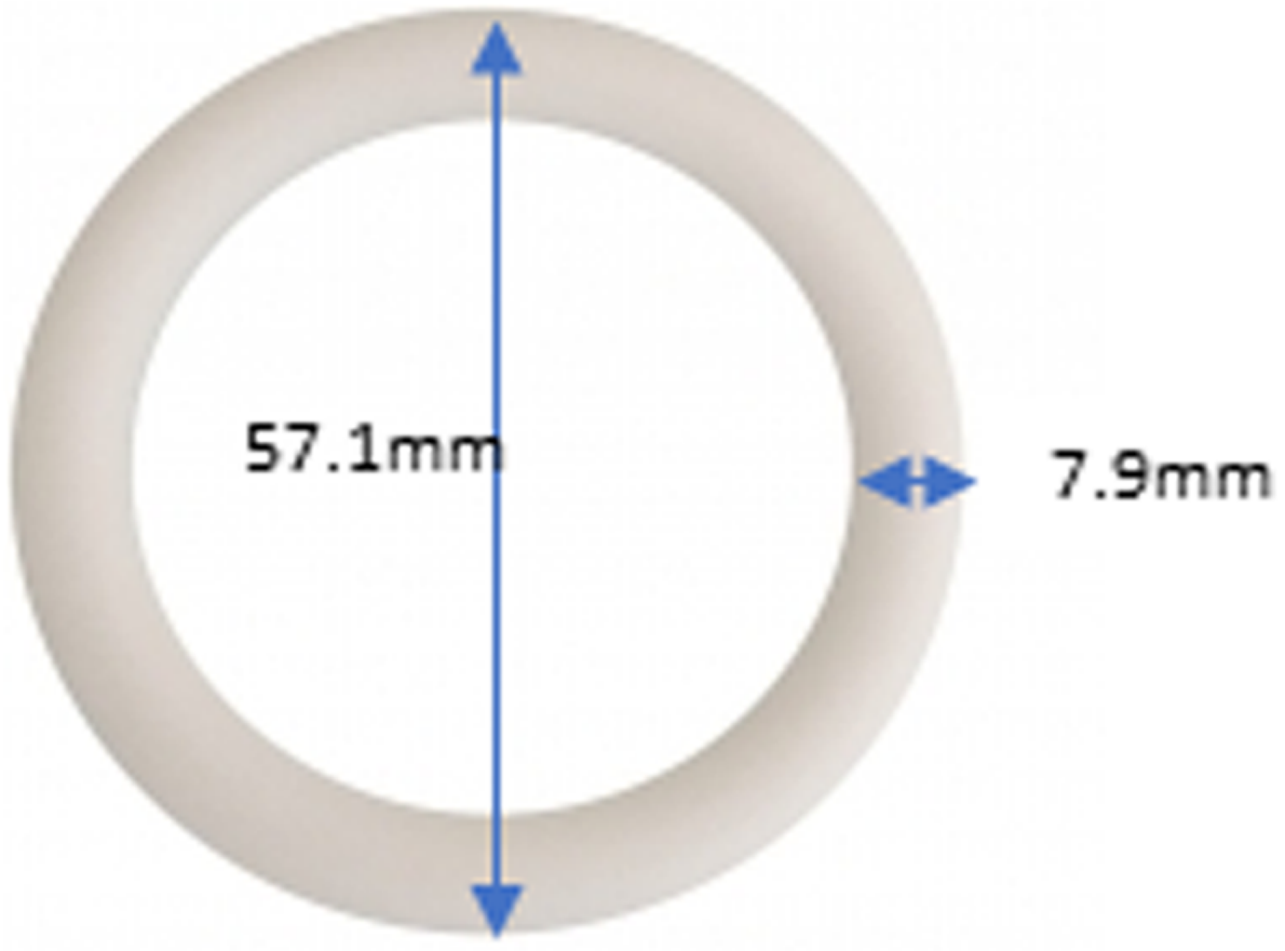
Study ring dimensions.

This 3-month MPT vaginal ring was a silicone matrix vaginal ring measuring 57.1 millimeters in outer diameter and 7.9 millimeters in cross-sectional diameter (see Figure 1). It contained 200 mg of dapivirine (for HIV prevention) and 320 mg of levonorgestrel (for contraception) and was developed by the International Partnership for Microbicides (IPM).

### Data collection

At enrollment [and after learning initial information about the study product – more details can be found in the Informed Consent form found in the study protocol (52) and the Study Specific Procedures manual (53)], all participants completed an acceptability questionnaire that included questions about prospective ring acceptability and initial concerns related to the study ring. Participants also completed an acceptability questionnaire at their Product Use End Visit (PUEV) that included questions about retrospective ring acceptability during the trial, experiences with ring use, and product preference. The questionnaire was derived from an earlier MPT ring study (54). Questions pertained to ease of use, ease of insertion, ease of removal, awareness and comfort of the ring, checking for ring presence, acceptability of changes in vaginal bleeding patterns, and how bothersome participants found any vaginal dryness or wetness from ring use. Product preference questions included preferences for HIV prevention methods, contraceptive methods, and separate vs. combined methods. The questionnaire also contained open ended questions about the participant's experience with the ring. Throughout their enrollment, participants were sent daily text messages regarding any changes in bleeding, and weekly text messages about any instances of their ring being removed or falling out (partially or fully).

All enrolled participants completed a qualitative in-depth interview (IDI) in English at PUEV, which occurred at Day 90 for all but four participants who terminated study participation early (but did complete an IDI before exiting the study). The IDI was conducted remotely using BlueJeans Video Network software by female social scientists with training in qualitative interviewing. Interviewers were based at RTI International (San Francisco and Berkeley, CA). The interviewer followed a semi-structured questionnaire guide to elicit participant experiences and opinions on study ring use, acceptability, adherence, and product preference. The interviews with 25 participants ranged from 28 to 88 min (average length of 50 min). Using notes taken during the IDI, the interviewer completed a debriefing report that summarized salient topics. The debriefing report was reviewed by another qualitative researcher and, upon finalization, shared with the protocol team. These reports provided prompt feedback and an opportunity to refine areas for probing in subsequent study interviews. Interviews were audio recorded and transcribed verbatim, and the resulting transcripts were reviewed for quality, finalized, and certified by the transcriptionist and project coordinator. Examples of questions in each of these data collection instruments are shown in Table 1.

**Table 1:** Example questions from relevant data collection instruments, presented by sequence of procedures.

Baseline acceptability questionnaire (Day 0)	• How worried are you about using one vaginal ring for 3 months? ○ *Very worried, Somewhat worried, A little worried, Not at all worried* • Overall, how much do you like the ring? ○ *Dislike very much, Dislike, Like, Like very much*
Daily text message (bleeding)	• Since your last SMS survey or clinic visit, have you had any spotting or bleeding? ○ *No, Light bleeding/spotting, Moderate bleeding, Heavy bleeding*
Weekly text message (ring outage)	• Did your ring ever partially fall out? ○ *Yes, No* • Other than as instructed by study staff, was your ring ever fully out? ○ *Yes, No*
Follow-up acceptability questionnaire *Also see* Table 3 (Day 90)	• The [first/last] time you inserted the ring in your vagina, was it difficult or easy to insert? ○ *Very difficult, Difficult, Easy, Very easy, I never inserted the ring* • Overall, how did it feel to have the ring inside you every day? ○ *Very comfortable, Comfortable, Uncomfortable, Very uncomfortable*
In-depth Interview (Between Day 90 and Study Exit)	• When you first learned about the ring, what kinds of concerns did you have? • What is your opinion about wearing the ring when having sex? • Other than the specific times you were asked to remove the ring by study staff, when was your ring removed? • Given the options of having a product like the study ring that provided 2-in-1 protection and having two separate products – one for each kind of prevention – how would you decide what you prefer?

### Data analysis

The qualitative data were analyzed thematically (55). Of the three qualitative data coders on the analysis team, two had also conducted the interviews with study participants. Two interviewer-coders came from sociobehavioral public health research backgrounds, and the third coder came from a clinical trial research background. All members of the analysis team were female and employed at an institution separate from that of the clinical trial and the product development teams. Quantitative data from the acceptability questionnaires and text messages were housed and managed at the Statistical Center for HIV/AIDS Research and Prevention (SCHARP) at Fred Hutchinson Cancer Research Center. These data, along with the IDI qualitative data, were analyzed at RTI International. The quantitative data were tabulated using Stata 17.0 (StataCorp LLC, College Station, Texas, USA).

The analysis team adapted codebooks from similar studies (MTN-036, MTN-038), incorporating constructs from the Theoretical Framework of Acceptability (56). All three coders on the analysis team applied a draft of the codebook to copies of the same study transcript to identify completeness of the codes and appropriateness of the definitions. Following this step, the codebook was updated and finalized. The coding team completed three sequential rounds of coding review, whereby each coder would review another coder's code application to a transcript. Coders met weekly to discuss questions that emerged, interesting findings, and reconcile any discrepancies identified in the coding review process. The transcripts were coded using Dedoose version 9.0.78 (SocioCultural Research Consultants, LLC, Los Angeles, CA, USA). Once coding was complete, code reports were generated to understand participants' positive and negative experiences that may have influenced adherence and their view of how acceptable the study ring was (codes included in code reports: Concern, Enabler, Future/Hypothetical, 2-in-1, Suggestions, Bleeding, Side effects/Safety, Shift/Expulsion).

All three coders contributed to writing summary memos that detailed the excerpts included in the code reports, the key messages conveyed, and themes and trends identified. The lead analyst used code reports and summary memos to develop a matrix table that documented each participant's overall assessments of the study ring, as well as salient experiences during their study participation. This matrix table was used to group women into how willing they would be to use this MPT ring in the future and analyze use experiences within those groups. This also allowed for re-categorizing participants by their assignment to the continuous or cyclic use regimen to examine any trends of use experiences within those groups.

The study protocol was approved by Advarra and the University of Pittsburgh IRBs. This study was collaboratively overseen by the Microbicide Trials Network (MTN), IPM, and the *Eunice Kennedy Shriver* National Institute of Child Health and Human Development (NICHD) of the National Institutes of Health (NIH). All participants provided written informed consent prior to any data collection activities.

## Results

### Participant demographics and prior use of contraceptive and vaginal products

Participants ranged in age from 21 to 43 years, with a median age of 36. The majority of participants were white, and two thirds held at least a college degree. The contraceptive methods used in the 30 days prior to study participation were male condoms (*n* = 10), non-hormonal intrauterine devices (*n* = 5), having a male partner who was sterilized (*n* = 3), fertility awareness (*n* = 2), withdrawal (*n* = 1), and/or female sterilization (*n* = 1). All participants had prior experience with vaginal insertion of a product such as a tampon, lubricant, sex toy, vaginal medication, menstrual cup, vaginal ring, or douche. Further descriptive characteristics of the study participants and their lifetime use of contraceptive methods and vaginally inserted products are provided in Table 2.

**Table 2:** Selected characteristics of study participants at enrollment.

	*n* = 25	%
Age in years [median (min - max)]	36	(21–43 years)
Hispanic or Latino	2	8
Race (mark all that apply)		
Asian	2	8
Black or African American	3	12
White	20	80
Highest level of education level completed		
High school graduate	4	16
Partial college	4	16
College graduate	8	32
Partial graduate school	3	12
Graduate school degree	6	24
Relationship Status		
Not in a relationship, single	10	40
In a relationship, not married	8	32
Married	6	24
Divorced	1	4
Currently has a primary sex partner	15	60
*Gender of primary sex partner* (*n* = 15)		
Man	13	87
Woman	1	7
Transgender man	1	7
Study product assignment		
Continuous (One 90-day cycle)	12	48
Cyclic (Three 28-day cycles with two-day removals periods)	13	52
Prior use of contraceptive methods*		
Male condom	25	100
Oral contraception	15	60
Emergency contraception	11	44
Contraceptive patch	3	12
Depot medroxyprogesterone acetate	6	24
Contraceptive/hormonal vaginal ring	4	16
Spermicidal sponge, foam, cream, or jelly	4	16
Intrauterine device	8	32
Implant	2	8
Withdrawal	16	64
Fertility awareness-based methods	9	36
Female sterilization	1	4
Male sterilization	3	12
Other [spermicidal suppository, assumption of male infertility]	2	8
Prior use of non-contraceptive vaginal products		
Vaginal medication in cream or gel form	12	48
Douche/vaginally applied “hygiene” product	6	24
Tampon	24	96
Menstrual cup	10	40
Personal or sexual lubricant	17	68
Sex toys	15	60
Other [Water]	1	4

*All participants reported prior use of at least one contraceptive method. No participants reported exposure to the female or internal condom or cervical barriers.

### MPT ring preferences

After three months of using the study ring, participants quantitatively reported a variety of experiences with half of participants each reporting that they liked it or liked it very much (*n* = 13, compared to *n* = 19 at baseline) or disliked (*n* = 12, compared to *n* = 6 at baseline) the MPT ring. In qualitative interviews, participants generally reported positive reactions to an MPT ring in theory yet reported that this version of the MPT ring had several flawed features. When discussing the idea of an MPT ring, participants were consistently enthusiastic about the possibility of a single product providing simultaneous prevention of HIV and unintended pregnancy: this was seen as convenient and decreasing the burden on the user. The vaginal ring delivery mechanism – especially if the duration was for 90 days – was viewed to decrease user burden and the risk of user error. Having a self-inserted product was also seen as advantageous as it does not require a medical provider to administer, thereby reducing the need for repeat clinic visits. Finally, participants also appreciated that use of a vaginal ring was discreet and controlled by the user. When asked quantitatively to compare the study ring to male condoms as a method of HIV prevention or contraception, 80% of participants liked the ring at least as much as condoms. Regarding their preference to use a single or dual-purpose method for contraception and HIV prevention, an overwhelming majority (88%) preferred a combined method (Table 3).

**Table 3:** Comparison of study ring to male condoms as reported in the 3-month follow-up acceptability questionnaire.

	*n* = 25	%
As a method to prevent HIV, which do you prefer to use - the ring or the male condom?		
Ring	7	28
Condom	4	16
Neither - I dislike both study products	1	4
Both - I like both study products equally	13	52
As a method OF CONTRACEPTION, which do you prefer to use - the ring or the male condom?		
Ring	10	40
Condom	4	16
Neither - I dislike both study products	1	4
Both - I like both study products equally	10	40
Would you prefer to use separate methods for contraception and HIV prevention or a combined method?		
Separate methods	0	0
A combined method	22	88
Don’t know	1	4
Don’t care	2	8

### Experiences with the study ring

Participants who reported negative experiences with use of this study ring described changes in vaginal bleeding (*n* = 17), perceived side effects (*n* = 10), and discomfort with the positioning of the ring in the vagina (*n* = 21). The impact of these challenges on their daily lives, in concert with individual willingness to navigate these challenges, led to a range of reported attitudes towards the vaginal ring. The most important challenges in determining participant views of the “real life” usability of the ring were: (1) more frequent or irregular ring-associated vaginal bleeding, (2) other perceived side effects (e.g., discharge, yeast infections, bacterial vaginosis, headaches, dizziness, acne, weight gain, decreased libido, vaginal odor, depression, mood swings) and (3) experiences of the ring slipping or falling out completely (partial or complete expulsions).

The 25 participants fell into three groups based on themes that emerged from this qualitative analysis. The first group consisted of a minority of participants (*n* = 3) who had minimal negative experiences with the study ring and concurrently stated they would use it in its current form. However, most participants experienced challenges in their day-to-day lives significant enough to negatively affect their willingness to use the ring. Among them, the second group (*n* = 14) found the product to be desirable in some ways and would consider using it in the future under certain conditions (i.e., fewer ring expulsions, less undesirable changes in bleeding, higher perceived individual HIV acquisition risk). The third group (*n* = 8) found that using the study ring was so problematic that they would never be interested in using it. Marital status was associated with the three groups, a demographic trend that would need to be further explored. Participants in the first group who were most willing to use the ring were all single, while being married was associated with the third group of participants unwilling to use the ring.

#### Willing to use the study ring (*n* = 3/25, 12%)

In qualitative in-depth interviews, 3 participants discussed interest in using this MPT vaginal ring in a “real life” situation. They did not report any perceived negative side effects associated with using the study product, and all experienced less vaginal bleeding, which was described as an unanticipated benefit of using the study ring. Light spotting was experienced by two of them and was reported as not outweighing the overall benefits of lighter/discontinued monthly periods. This group reported mixed experiences with the ring slipping and none of them experienced complete expulsion of the ring.

**Figure 2:**
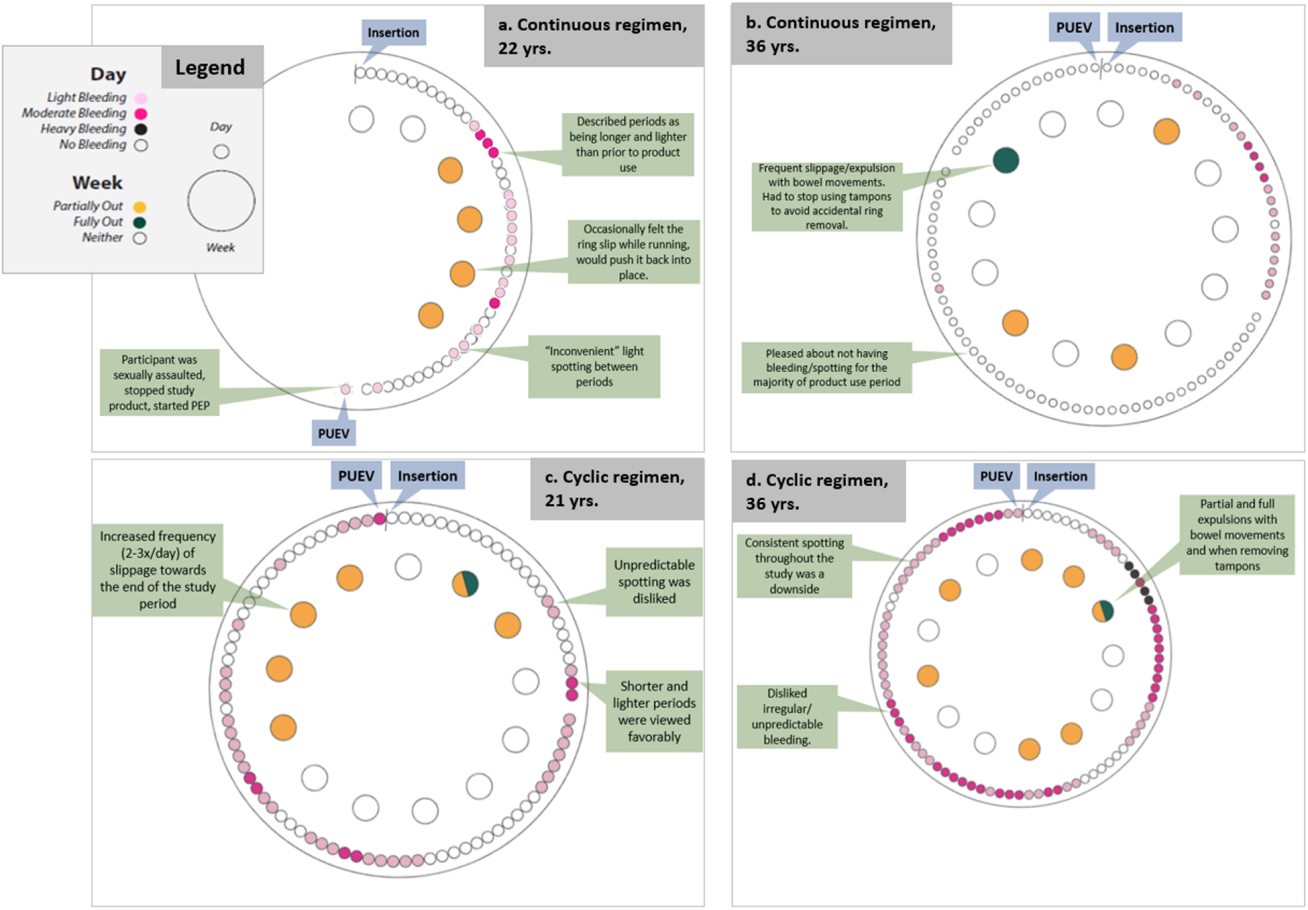
Participant reports of bleeding and slippage/expulsion: four illustrative cases using data from qualitative interviews and SMS reports.

One of these three participants (see Figure 2A) was sexually assaulted while using the MPT ring. This participant was unique in that she experienced a transient increase in her personal risk for HIV acquisition, saying, “*it's now very real to me that this is a very real, like, condition that I can get*.” She reported that she would have liked to use the ring even if it were just a contraceptive product, but after the sexual assault, she felt that the added HIV indication was also very important. She said, “*If the ring works as like a birth control, that would be just enough for me, but the like HIV part is like huge now that I've experienced what I have*” (age: 22, regimen: continuous).

#### Would use the study ring, with caveats (*n* = 14/25, 56%)

Over half of the participants (*n* = 14) reported they would consider possible use of this MPT ring in the future, if improvements were made to the ring. Many of these women reported that they had significant experiences with the ring (e.g., spotting in between or instead of menses; side effects like acne, discharge, yeast infection, changes in vaginal bleeding, vaginal odor, depression and anxiety; and/or challenges with slippage and expulsion) that interfered with daily activities and that were not acceptable given their low-risk category. These participants also reported willingness to use this ring – in its current form – were they to be at higher HIV risk. A few participants noted that since this is an MPT, a person would have to perceive themselves at high risk for HIV and be highly motivated to prevent pregnancy to be willing to manage the downsides of this MPT ring.

One of these participants (see Figure 2B) reported this MPT ring as “so great,” particularly the long-acting characteristic and the ability to self-administer. After insertion she experienced some bleeding which soon stopped. For the remainder of the study, she experienced no spotting or bleeding which she “loved” and none of the negative side effects she had experienced with other hormonal contraceptives (i.e., acne, changes in mood, unpredictable bleeding). However, her experience with frequent partial and full expulsions ultimately would have driven her away from using this MPT ring. She said, “*I think it's so great to have two of these products in one, and that my only, my only caveat in it would be, would be it falling out. That was just, if there's some way to tweak that I think this would be, this would be fantastic*” (age: 37, regimen: continuous).

Another participant (see Figure 2C) reported a more negative experience with use of the ring. Towards the end of the study, her ring frequently slipped out of place. She also reported the ring as unpleasant to use during sex and that she experienced unpredictable spotting and a noticeable vaginal odor with ring use. This participant also reported her perspective that someone who was at higher risk would find that the benefits of using this ring (extended duration, low opportunity for user error, shorter and lighter monthly periods) would outweigh the downsides.

#### Would not use the study ring (*n* = 8/25, 32%)

Eight participants reported that they would not use this ring, even if they were at higher HIV risk. These participants reported that either the ring itself was unusable or the ring was incompatible with their body. A subset of these participants (*n* = 3) hypothesized about changes to the ring that would lead them to reconsider. Though these women did not think this ring was “right” for them in its current form, many continued to like the MPT ring in theory, and thought it could be right for others who had higher HIV risk or who did not experience as many challenges.

Most of the women who said that they would not use this ring – at least in its current form - reported issues with vaginal bleeding and/or the ring staying in place. Unpredictable breakthrough bleeding, heavy and painful periods, and/or prolonged bleeding were reported among 5 of these 8 participants and were seen as unacceptably disruptive to these participants' lives. Multiple participants (7 of the 8 in this group) reported that the ring frequently slipped out of place and was on the verge of complete expulsion during bowel movements, causing the user to need to hold it in place or re-position it daily or more frequently. Participants who experienced regular sensations of ring slippage reported a mental burden associated with constant worry about expulsion and about needing to re-position the ring.

A participant (see Figure 2D) who experienced both constant bleeding and frequent slippages liked some aspects of the ring (dual indications and the fact that one could, in theory, “insert it and forget it”), though she reported that she spent mental energy worrying about the ring falling out whenever she had a bowel movement and tried to plan her day so she would be home for bowel movements rather than deal with public restrooms. Even though she typically experienced heavy flow and strong cramping during her period prior to ring use, she thought that was preferrable to the “annoying” ongoing spotting while using the ring. She also experienced three urinary tract infections during the study which was atypical for her. Together, these factors led her to state that she would not consider using this ring in the future.

#### Perspectives on continuous vs. cyclic regimen

Participants who were randomized to continuous ring use conveyed more positive experiences compared to those randomized to cyclic use (Table 4), with 75% of continuous users and 58% of cyclic users expressing willingness to use in the future (with or without caveats). Two of the three participants who reported willingness to use the ring in its current form were assigned to the continuous regimen, and the third participant was assigned to the cyclic regimen but appeared to view herself as a continuous user^a^. Of those who were willing to use with caveats, half were assigned to the continuous regimen. Of those who reported unwillingness to use the ring at all in its current form, a majority (5/8; 63%) were randomized to the cyclic use regimen.

**Table 4:** Willingness to use and regimen preference, by assigned use regimen (continuous vs. cyclic) as reported in the follow-up acceptability questionnaire.

	Assigned to continuous use regimen (*n* = 12)	Assigned to cyclic use regimen (*n* = 13)
Level of willingness to use in the future		
Willing to use	2	1^a^
Would use, with caveats	7	7
Would not use	3	5
Regimen preference		
Continuous use	10	6
Neutral	2	3
Cyclic use	0	4

^a^ This participant was randomized to the cyclic regimen and used the ring cyclically per clinical records. However, during her interview she referred to herself as among the group of continuous users, stating that she “did have it in continuously for ninety days.”

Of the 12 participants assigned to continuous use of the ring, there was a strong preference for continuous use if given the option (10 preferred continuous, 2 were neutral). When describing their motivations, participants cited a continuous use regimen as affording greater peace of mind, saying, “…*leaving it in is probably better because with any user-dependent [laughter] method, like the less thing the user has to do, the less prone to error it is*” (age: 29, regimen: continuous). These participants also thought that removing it wouldn't make any difference but had concerns about the logistics of removing and reinserting it. One participant said, “*I prefer continuous, the less I have to worry about taking things out and remembering to put it back in, the better*” (age: 36, regimen: continuous).

The 13 participants assigned to the cyclic regimen reported mixed opinions on use regimen preference. Six of them (46%) expressed a preference for continuous use, 4 (31%) favored cyclic use, and 3 (23%) were neutral. Those in the cyclic regimen who expressed interest in continuous use cited anticipated convenience, avoidance of extra health facility visits (if required for each removal/insertion), and avoidance of necessary logistics to store the ring during the 2-day removal period (i.e., refrigerating it sanitarily and at home) as reasons for their expressed preference. Of the 4 participants who preferred cyclic use, three cited having a “break” from the worry about it slipping/falling out as the predominant rationale. Other reported reasons for favoring cyclic use included a perception that the 2-day removal period may have aligned with lighter bleeding and that removing the ring would offer a scheduled opportunity to check on the ring, as one participant said: “*I don't like the idea that you're just going to forget about it for months at a time*” (age: 43, regimen: continuous).

#### Feedback to the product developer

Ongoing challenges with vaginal bleeding issues, recurrent ring slippage, and expulsions with this version of the MPT vaginal ring were regularly reported and discussed with IPM (the product developer) throughout the life of the study. The product developer was part of the study management team, allowing for frequent conversations where emerging themes from the qualitative data could be discussed alongside updates from the clinical trial team. This informed the design of the next iteration of the MPT ring currently being evaluated in the US (NCT05041699). The dapivirine vaginal ring has a considerably higher Shore score (with increasing Shore score reflecting increased hardness), compared to the study MPT ring used in the MTN-044 study (57). A common concern of participants in dapivirine vaginal ring clinical trials was the rigidity of the ring. For the MPT ring used in this study, the addition of levonorgestrel to the matrix ring formulation resulted in a softer ring that may have increased the rate of slippages compared to the stiffer DPV-only ring. Though the softness of this MPT ring may help to address prior user concerns about the firmness of DVRs expressed previously, the more supple quality of this MPT ring relative to other prior vaginal rings may be inadvertently at the expense of ring retention in the vagina. Due to these challenges, modifications to the product formulation have subsequently been undertaken, and the Shore score of the re-formulated ring is similar to that of other vaginal rings approved for use by regulatory authorities.

## Discussion

Women in the MTN-044/IPM 053/CCN019 Phase I trial in the United States favored the concept of an MPT vaginal ring for simultaneous prevention of HIV and unintended pregnancy. Though the study ring was well-tolerated during the clinical trial based on a low rate of discontinuation (48), most participants reported that they would not want to use this version of the MPT ring due to challenges they experienced with the ring during the study. However, most participants also expressed interest in the study ring if their personal risk for HIV and/or motivation to prevent pregnancy increased in the future, or changes were made to the ring that would result in fewer expulsions or less vaginal bleeding. Participants approved of an MPT ring that would be easy and convenient to use for 3 months, thereby decreasing user burden and preventing unnecessary user error. Ideally, the ring could be initiated, administered, and controlled by the user and thus reduce repeated clinical visits with a medical provider.

However, participant views of the MPT ring used in the study were more varied, with more positive views expressed by those who experienced fewer challenges (slippages, expulsions, side effects, changes in bleeding) and those who were assigned to the continuous regimen. Compared to previous research where participant opinions of products increased after use (13, 40), it is notable that participants in this study reported liking the ring less at follow-up than at baseline. This highlights two important reflections: First, the challenges that participants encountered with using this ring dampened the original enthusiasm that the participants had (all of whom had experience with vaginally inserted products). Second, this supports the finding in other studies which suggested the positive change in attitude after exposure was a reflection of overcoming initial apprehension and finding the product more desirable than at baseline, rather than due to social desirability. These findings are useful to understand nuances of user preferences for an MPT ring, to inform future MPT study designs and reformulation of next generation MPT rings, and to encourage deeper discussion about the relationship between consumers' increasing desire for a perfect prevention product and an effective product with limitations.

Although many study participants saw how an MPT ring could be an easy-to-use, convenient, user-controlled option, the majority of the participants were not interested in using the study ring in its current form. About 1/3 of participants stated that some characteristics of the vaginal ring would have to change to make it usable for them. Dissatisfaction with the ring came from three main issues: unanticipated, heavy, or prolonged bleeding; ring slippage and expulsion; and other perceived side effects such as weight gain, acne, and changes in vaginal discharge/odor. Future MPT studies would benefit from exploring the people's willingness to manage unscheduled bleeding and other side effects as factors influencing ring acceptability.

When determining whether the ring was a good “fit” for them (or someone else), one of the prevailing caveats that women shared was the user's self-perceived risk for both HIV and unintended pregnancy. It is important to note that women in this study had – by requirement – low risk for both; thus, they often contrasted their own willingness to use this product with others who may have higher risk levels, or with hypothetical situations they judged to be riskier. Many of them hypothesized that they would use the ring if they felt more at risk for HIV infection and unintended pregnancy. To better tease out the relationship between participants' perceived risk levels and their willingness to use the ring, it is essential for future acceptability research to include women with various life contexts and needs for prevention of HIV and pregnancy.

In this trial, user perspectives reflected a preference for continuous (rather than cyclic) use: participants assigned to the continuous regimen reported more positive experiences, and most participants – regardless of study arm assignment – reported preferring a continuous ring for hypothetical future use. The smaller portion of participants who preferred cyclic use largely cited a desire to take a “break” from bleeding/slippage related challenges, rather than proactively preferring a cyclic regimen. Though the continuous regimen was preferred primarily for its convenience and less user burden, it is worth pointing out that making a choice in real life may be more complex. First, these women only used one regimen, thus lacking first-hand experience with both regimens to make a direct comparison. Therefore, if feasible, future studies may consider a crossover design to allow participants experience both regimens for individual-level comparison. Secondly, the prevention efficacy of either regimen, in addition to the ring's pros and cons, will need to be factored in decision-making. Cyclic use may represent an opportunity for user error (loss/damage, forgetting to replace, etc.) which could compromise effectiveness. Additionally, while a cyclic product has been traditonally used in contraception to allow withdrawal bleeds, the impact of periodic ring removal on HIV prevention efficacy is not well understood. While a longer period of ring removal in a cyclic regimen may be necessary to see an improvement in bleeding patterns (58), this is constrained by the need to maintain protective dapivirine levels. This underscores the importance for future research to focus on elucidating the pharmacokinetic and pharmacodynamic ramifications of ring removals so that women can make informed decisions based on the timing of ring insertions and removals relative to sexual exposures. As indicated by the minority who preferred the cyclic regimen who appreciated the chance to take a “break” from product use, MPT ring users may appreciate the opportunity to decide if and when to do event-based ring removals (e.g., for a sexual encounter, to wash the ring, in certain cases of pregnancy ambivalence) if periodic removals do not affect efficacy.

Over decades of development for reproductive health prevention products, particularly contraceptive products for women, the bar for achieving high product acceptability has been gradually raised. The current generation of reproductive health prevention products research for all genders has a refreshing and inspiring push for products to be *desirable* – not merely tolerable, or even acceptable (59). With this evolution of standards for biomedical prevention, and with multiple options becoming a reality (beyond the current contraceptive method mix), consumers of health products rightfully have a lower tolerance for undesirable side effects and negative impacts on their day-to-day lives. The data in this study with low-risk participants suggests that women who found MPT ring use to be relatively unobtrusive (lighter bleeding, few side effects) also found the benefits of HIV and pregnancy prevention (and possibly reduced menstrual bleeding) appealing, as there were no real downsides to counterbalance them. However, for users who encountered challenges (increased bleeding, slippage, and other side effects) in their daily lives, the product would need to present very strong benefits to outweigh any negative experiences and be deemed worthwhile. Even women at higher risk for both HIV acquisition and unintended pregnancy could judge a product as acceptable in short research studies, but excessive unfavorable side effects could sway them away from continued use – despite their risks – and present a barrier to real-world uptake and/or adherence. While high perceived risk of HIV acquisition and unintended pregnancy may be a facilitator to acceptability of a MPT vaginal ring, women often underestimate their actual risk (60–62). Therefore, a particular strength and opportunity for an MPT vaginal ring may be the ability to frame it positively as a more holistic tool that helps users optimize their sexual health, rights, and pleasure, as suggested in the “triangle approach” presented by Gruskin et al. (63), rather than using risk-based messaging which can be perceived as judgmental and discriminatory.

### Strengths and limitations

The MPT ring in this study is the first iteration of a dual-purpose preventive vaginal ring. As the first clinical trial in which participants compared cyclic and continuous use of a co-formulated contraceptive/HIV preventative MPT vaginal ring over 90 days, this study provides insights into factors that resulted in a wide range of acceptability related to side effects associated with the contraceptive indication, ring retention, and use of the ring cyclically (2-day removals every 28 days) vs. continuously for 90 days. These factors will help to inform future MPT ring development and acceptability research design.

An important limitation of this study is the participants' low likelihood for both pregnancy and HIV acquisition at baseline: only 13 of the 25 participants (or 52%) were sexually active with a male primary partner. Low likelihood of HIV acquisition and pregnancy was an intentional feature of the eligibility criteria for the Phase I trial, yet it limits our ability to understand how women in different circumstances relative to HIV and/or unintended pregnancy may have differed in weighing the indication-related benefits of the ring against the challenges experienced with study product use. Many study participants posited that they might have felt differently about the study ring had they been at higher risk of HIV and/or pregnancy. It will be important to pursue further acceptability research with women with varying prevention needs in next-stage clinical trials of the second generation of this MPT ring. By design, our sample size was small (*n* = 25). The sample also lacked diversity (single-site study, all participants were recruited in Pittsburgh, Pennsylvania, USA, and the study sample had relatively high levels of education and limited racial and ethnic diversity). By nature of the recruitment and enrollment process, all participants agreed to participate in a study where they knew they would be using a vaginal ring, indicating receptivity to the idea of vaginal ring usage prior to study product initiation. Additionally, all participants had prior experience with vaginally inserted products. The increase in participants reporting at follow-up (from baseline) that they disliked the ring suggests that the familiarity or comfort with vaginal product usage did not necessarily translate into comfort with this product.

## Conclusion

Participants in this study had positive reactions to MPT rings in theory, yet the study MPT ring raised several concerns related to user experience and product acceptability. Though all participants understood and appreciated the benefits of a woman-initiated, longer-term, MPT product, this sample of low-risk participants found changes in vaginal bleeding, the ring slipping/falling out of place, and other side effects problematic. Importantly, these study findings contributed to development of the next generation of this MPT ring. Conducting qualitative research with participants during early-stage clinical trials can offer critical design modifications to product developers that may help to improve the future success of biomedical technologies.

## Data Availability

The raw data supporting the conclusions of this article will be made available by the authors, without undue reservation.

## Footnotes

^a^This participant was randomized to the cyclic regimen and used the ring cyclically per clinical records. However, during her interview she referred to herself as among the group of continuous users, stating that she “did have it in continuously for ninety days.”
